# The complete chloroplast genome sequence of *Camellia granthamiana*

**DOI:** 10.1080/23802359.2019.1692703

**Published:** 2019-11-20

**Authors:** Zhenzhong Jiang, Peng Jiao, Zhuo Qi, Jing Qu, Shuyan Guan

**Affiliations:** a College of Life Sciences, Jilin Agricultural University, Changchun, China;; b College of Agronomy, Jilin Agricultural University, Changchun, China

**Keywords:** *Camellia granthamiana*, chloroplast genome, phylogenetic analysis

## Abstract

*Camellia granthamiana* is a rare and endangered plant peculiar to China, and a total of 5 plants have been found at present. Based on the next generation sequencing, the whole chloroplast (Cp) genome of (Camellia granthamiana Sealy) of Camellia oleifera was constructed.In this study, the complete chloroplast (cp) genome of *Camellia granthamiana* was assembled based on next generation sequencing.The cp genome was 157,001 bp in length, including a large single copy (LSC) region of 70,387 bp, a small single copy (SSC) region of 18,296 bp and a pair of inverted repeats (IRs) of 52,082 bp. The genome contained 135 genes, including 90 protein-coding genes, 37 tRNA genes and 8 ribosomal RNA genes. The majority of these gene species occurred as a single copy.

*Camellia granthamiana* (*Camellia granthamiana Sealy*) Camellia,family to evergreen small trees in Hong Kong and Guangdong, discovered by Sealy in 1956(yongfu Yu.1999). Only one strain was found at the time of discovery, followed by adjacent Ma on Shan in the New Territories of Hong Kong and Lianhua Mountain in Haifeng, Guangdong Province. Its model specimens were collected from Dayu Mountain on the Kowloon Peninsula, and only two clusters of plants were found. It is distributed in the wet rock seam of the local valley of Guangdong, but between 4 and 5, it is included in the national secondary protection plant. *Camellia granthamiana* was only made public in 1956, including only two shrubs from the model producing area, and only 4 or 5 bushes have been found, which are in danger of being destroyed at any time and must be protected. *Camellia granthamiana* is in IUCN Red list-list of extremely dangerous and endangered plants in China in word (Katoh and Standley 2013; Lohse et al. 2013) (Figure 1).

**Figure 1.**
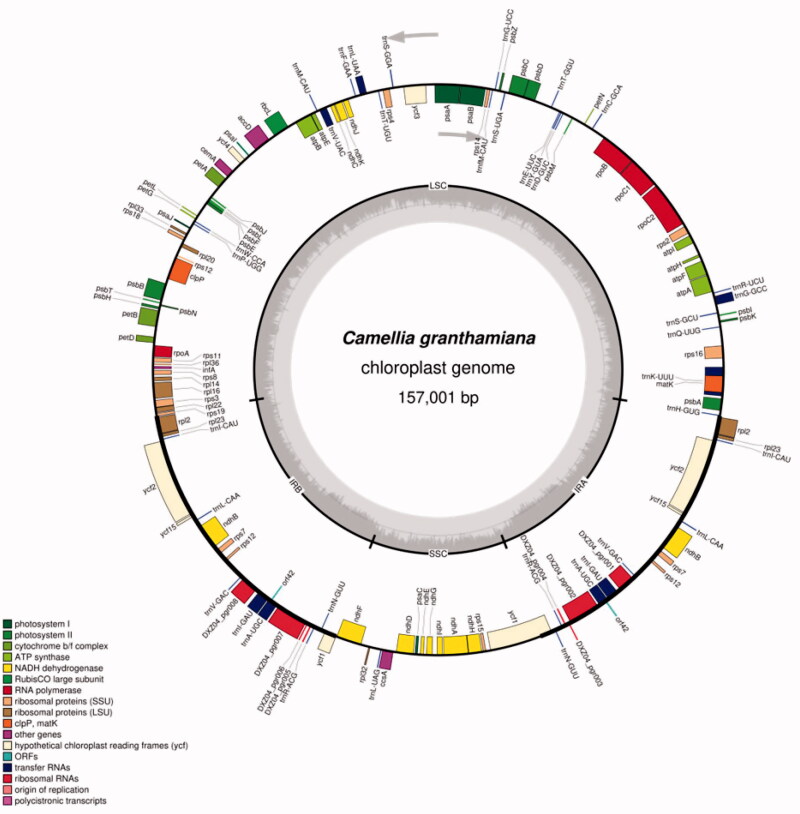
Gene map of the Camellia granthamiana chloroplast genome.

To ascertain the phylogenetic position of *Camellia granthamiana*, we conducted a phylogenetic analysis usingtwenty-two com- plete chloroplast genomes including*Camellia reticulata, Camellia ptilophylla., Actinidia ulmifolia C. F. Liang, Actinidia chinensis Planch., Lonicera japonica Thunb., Panax ginseng C. A. Mey, Panax notoginseng (Burkill) F. H. Chen ex C*. H.Etc. (Kearse et al. 2012; Shaw et al. 2014) (Figure 2). The results showed that the three species were in the same branch. Dianshan tea is most closely related to big bract white camellia. This complete chloroplast genome can be easily used in population genome research of Camellia oleifera (Ronquist and Huelsenbeck 2003), and this information will develop new conservation rules and management practices for this endangered species.

**Figure 2.**
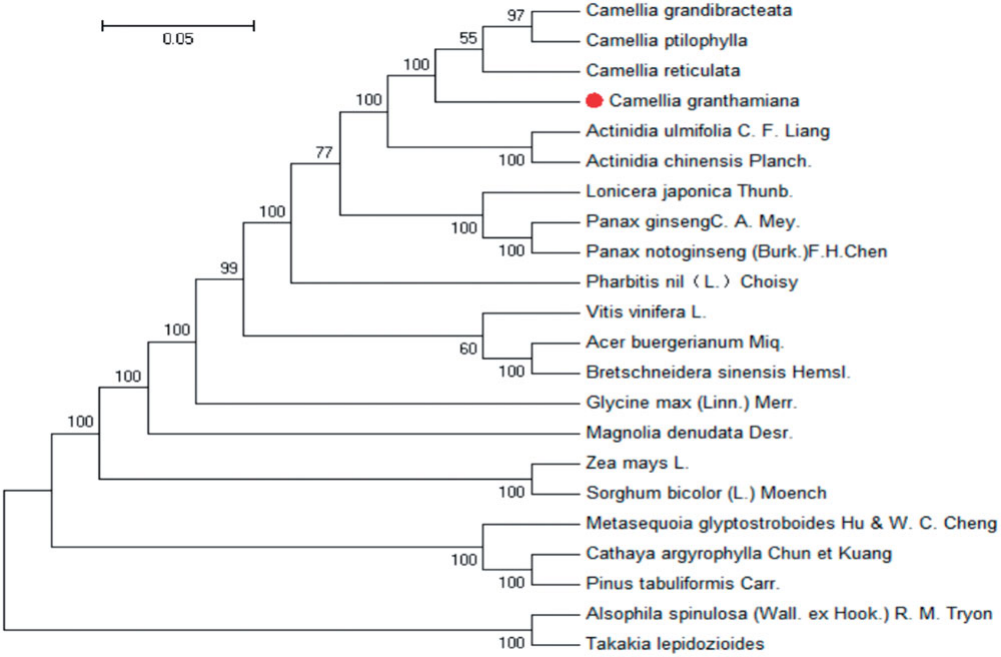
The phylogenetic tree based on the 22 complete chloroplast genome sequences. Bayesian posterior probabilities/ML boot-strap values are shown at nodes. Accession numbers: *Camellia grandibracteata* (NC_024659.1), *Camellia ptilophylla* (NC_038198.1), *Camellia reticulata* (NC_024663.1), *Camellia granthamiana* (NC_038181.1), *Actinidia ulmifolia C. F. Liang* (NC_031187.1), *Actinidia chinensis Planch*. (NC_026690.1), *Lonicera japonica Thunb.* (NC_039636.1), *Panax ginseng C. A. Mey* (NC_006290.1), *Panax notoginseng (Burkill) F. H. Chen ex C. H.* (NC_026447.1), *Pharbitis nil (L.) Choisy.* (NC_031159.1), *Vitis vinifera* (NC_007957.1), *Acer buergerianum Miq.* (NC_034744.1), *Bretschneidera sinensis* (NC_012818.1), *Glycine max(L.) Merr* (NC_007942.1), *Magnolia denudata Desr.* (NC_018357.1), *Zea mays L.* (NC_001666.2), *Sorghum bicolor (L.) Moench* (NC_008602.1), *Metasequoia glyptostroboides Hu & W. C. Cheng* (NC_027423.1), *Cathaya argyrophylla Chun et Kuang* (NC_014589.1), *Pinus tabuliformis Carrière* (NC_028531.1), *Alsophila spinulosa (Wall. ex Hook.) R. M. Tryon* (NC_012818.1), *Takakia lepidozioides* (NC_028738.1).

In this study, *Camellia granthamiana* was sampled fromWutongshan, Guangdong, China (13°17′∼114°18′E, 22°23′∼22°43'N). A voucher specimen (JLAU 0191004) was deposited in the Jilin Agricultural University, Jilin, China.
